# The pretreatment Controlling Nutritional Status (CONUT) score is an independent prognostic factor in patients with resectable thoracic esophageal squamous cell carcinoma: results from a retrospective study

**DOI:** 10.1186/s12885-016-2696-0

**Published:** 2016-09-06

**Authors:** Takahiro Toyokawa, Naoshi Kubo, Tatsuro Tamura, Katsunobu Sakurai, Ryosuke Amano, Hiroaki Tanaka, Kazuya Muguruma, Masakazu Yashiro, Kosei Hirakawa, Masaichi Ohira

**Affiliations:** 1 Department of Surgical Oncology, Osaka City University Graduate School of Medicine, 1-4-3 Asahimachi, Abeno-ku, Osaka, 545-8585 Japan; 2 Department of Gastroenterological Surgery, Osaka City General Hospital, 2-13-22, Miyakojimahondohri, Miyakojima-ku, Osaka, 534-0021 Japan

**Keywords:** Esophageal cancer, Esophagectomy, Prognostic factor, Nutrition, Controlling nutritional status

## Abstract

**Background:**

The purpose of this study was to investigate the impact of the Controlling Nutritional Status (CONUT) score on survival compared with the platelet to lymphocyte ratio (PLR), the neutrophil to lymphocyte ratio (NLR), and the Glasgow Prognostic Score (GPS) in patients with resectable thoracic esophageal squamous cell carcinoma (ESCC).

**Methods:**

One hundred eighty-five consecutive patients who underwent subtotal esophagectomy with curative intent for resectable thoracic ESCC were retrospectively reviewed. Time-dependent receiver operating characteristic curve analyses for 3-year overall survival (OS) as the endpoint were performed, and the maximal Youden indices were calculated to assess discrimination ability and to determine the appropriate cut-off values of CONUT, PLR, and NLR. The patients were then classified into high and low groups based on these cut-off values. Correlations between CONUT and other clinicopathological characteristics were analyzed. Prognostic factors predicting overall survival (OS) and relapse-free survival (RFS) were analyzed using Cox proportional hazards models.

**Results:**

The areas under the curve predicting 3-year OS were 0.603 for CONUT, 0.561 for PLR, 0.564 for NLR, and 0.563 for GPS. The optimal cut-off values were two for the CONUT score, 193 for PLR, and 3.612 for NLR. The high-CONUT group was significantly associated with lower BMI, high-PLR, high-NLR, and GPS1/2 groups. On univariate analysis, high-CONUT, high-PLR, high-NLR, and GPS 1/2 groups were significantly associated with poorer OS and RFS. Of these factors, multivariate analysis revealed that only the CONUT score was an independent prognostic factor for OS (HR 2.303, 95 % CI 1.191–4.455; *p* = 0.013) and RFS (HR 2.163, 95 % CI 1.139–4.109; *p* = 0.018).

**Conclusions:**

The CONUT score was an independent predictor of OS and RFS before treatment and was superior to PLR, NLR, and GPS in terms of predictive ability for prognosis in patients with resectable thoracic ESCC.

## Background

Despite significant improvements in the diagnosis and treatment of patients with esophageal cancer, their prognosis still remains poor due to its aggressive biological behavior [1]. Although surgical resection is the mainstay of treatment for local and locoregional disease in esophageal cancer, neoadjuvant treatment has been widely accepted as a means of improving the prognosis of esophageal cancer [2–5]. Therefore, predicting prognosis using pretreatment clinical variables, but not operative and pathological variables, is important to improve the prognosis and to offer an optimal treatment strategy.

There is accumulating evidence that the presence of a systemic inflammatory response and malnutrition are associated with a worse prognosis in various malignancies [6–9]. Recently, several inflammation-based markers, such as the platelet to lymphocyte ratio (PLR), the neutrophil to lymphocyte ratio (NLR), and the Glasgow Prognostic Score (GPS), have been reported to be prognostic factors in various malignancies, including esophageal cancer [10–15]. The Controlling Nutritional Status (CONUT) score, which is calculated by the serum albumin concentration, the total peripheral lymphocyte count, and the total cholesterol concentration, was developed as a screening tool for early detection of poor nutritional status [16]. Use of the CONUT score has some advantages, such as simplicity and cost effectiveness, but there have been few reports on the relationship between the CONUT score and clinical outcomes in malignancies [17]. Therefore, the significance of the CONUT score in the treatment of esophageal cancer is still unknown.

The aim of this study was to elucidate the impact of the pretreatment CONUT score on survival compared with other inflammation-based markers (PLR, NLR, and GPS) in patients with resectable thoracic esophageal squamous cell carcinoma (ESCC).

## Methods

The clinical data of consecutive patients who underwent subtotal esophagectomy with curative intent for resectable thoracic ESCC at Osaka City University Hospital (Osaka, Japan) between January 2000 and December 2014 were retrospectively reviewed. In this study, resectable thoracic ESCC was defined as patients without cT4 tumor and distant metastases on pretreatment examination. All patients were diagnosed with ESCC by biopsy before initial treatment. For reliable analysis, only thoracic ESCC patients who underwent two- or three-field lymphadenectomy and reconstruction using a gastric tube through the posterior mediastinum by cervical anastomosis were included. There was no uniform guideline for preoperative treatment until 2009; from 2009, neoadjuvant chemotherapy consisting of 5-fluorouracil/cisplatin or 5-fluorouracil/nedaplatin was administered for patients with clinical stage II/III in principle. Adjuvant chemotherapy was scheduled for patients with positive lymph node metastasis. Eight patients whose entire set of preoperative laboratory data was not available were excluded from this study. Ultimately, 185 patients were included. Forty-six patients received preoperative treatment; 39 patients received chemotherapy, 6 patients received chemoradiotherapy, and 1 patient received radiotherapy. This retrospective study was approved by the ethics committee at our institution and was conducted in accordance with the principles of the Declaration of Helsinki. Informed consent was obtained from all patients before treatment.

The pretreatment staging workup in principle included physical examination, laboratory tests, esophageal barium meal examination, upper GI endoscopy, enhanced computed tomography (CT) scans between the neck and upper abdomen, and positron emission tomography-computed tomography (PET-CT). On the basis of these examinations, tumor stage was assessed using the 6^th^ edition of the International Union Against Cancer [18]. Blood samples were obtained during the patients’ first visit to our institution before initial treatment. The CONUT score was calculated as described in Table 1. The PLR was calculated by dividing the platelet count by the lymphocyte count. The NLR was calculated by dividing the neutrophil count by the lymphocyte count. The GPS was constructed as follows: patients with both elevated C-reactive protein (CRP) (>1.0 mg/dl) and low albumin (<3.5 g/dl) were assigned GPS 2, those with elevated CRP (>1.0 mg/dl) or low albumin (<3.5 g/dl) were assigned GPS 1, and those with normal CRP (≤1.0 mg/dl) and normal albumin (≥3.5 g/dl) were assigned GPS 0.

**Table 1:** Scoring system for the CONUT

Parameter	Undernutrition degree			
	None	Light	Moderate	Severe
Serum albumin (g/dL)	≥3.50	3.00–3.49	2.50–2.99	<2.50
Score	0	2	4	6
Total lymphocyte count (/mm^3^)	≥1600	1200–1599	800–1199	<800
Score	0	1	2	3
Total cholesterol (mg/dL)	≥180	140–179	100–139	<100
Score	0	1	2	3
CONUT score = Serum albumin score + Total lymphocyte count score + Total cholesterol score				

Ignacio de Ullibarri J et al. Nutr Hosp [16]

Pretreatment variables, such as age, sex, body mass index (BMI), Eastern Cooperative Oncology Group performance status (PS), American Society of Anesthesiology score (ASA), tumor location, clinical TNM stage, and serum squamous cell carcinoma antigen (SCCA) levels were evaluated.

### Follow-up

The patients were followed every 3–4 months for the initial 2 years, every 6 months for the next 3 years, and annually thereafter. On a semiannual basis or on suspicion of recurrence, a clinical history was taken, and a physical examination, routine blood tests, measurements of SCCA, and enhanced CT scans between the neck and upper abdomen were performed. PET-CT was conducted if necessary. Recurrence was diagnosed according to the findings of these scheduled examinations. If the patients had not visited the hospital, follow-up information was obtained from telephone calls to the patients, family members, or their referring physicians.

### Cut-off determination and primary outcomes

To set the cut-off values for CONUT, PLR, and NLR, time-dependent receiver operating characteristic (ROC) curve analyses for 3-year overall survival (OS) as the endpoint were performed, and the maximal Youden indices were calculated [19]. All patients were classified into two groups based on these cut-off values. OS and relapse-free survival (RFS), which were the primary outcomes, were calculated from the start date of treatment to the date of last follow-up or death and to the date of confirmation of recurrence or death, respectively.

### Statistical analysis

Fisher’s exact test or the chi-square test was used for analyzing associations between categorical variables. Survival rates were calculated by the Kaplan-Meier method, and survival curves were compared with the log-rank test. Univariate analyses and multivariate analyses for OS and RFS were conducted with Cox proportional hazards models. To compare the prognostic value of each inflammation-based and nutritional marker, multivariate analyses including variables with *p* < 0.1 on univariate analyses and CONUT, PLR, NLR, or GPS, respectively, were performed, because CONUT, PLR, and NLR include the lymphocyte count, and CONUT and GPS include the albumin levels in their calculation. The hazard ratios (HRs) and 95 % confidence intervals (CIs) were calculated. A value of *p* < 0.05 was considered significant. These statistical analyses were performed with SPSS software (SPSS, Inc., Chicago, IL, USA), except for the time-dependent ROC curve-analyses that were performed with R-project Software, version 3.2.1.

## Results

### Time-dependent ROC curve-analyses

The time-dependent ROC analyses showed the areas under the curve (AUCs) predicting 3-year OS were 0.603 for CONUT, 0.561 for PLR, 0.564 for NLR, and 0.563 for GPS. The sensitivity and specificity of CONUT, PLR, NLR, and GPS were 19 % and 95, 26 and 87 %, 22 % and 92 %, and 16 % and 96 %, respectively. When the CONUT score was 2, PLR was 193, and NLR was 3.612, the Youden indices were maximal; therefore, these values were selected as the cut-off values.

### CONUT and patients’ clinicopathological characteristics

The patients’ median age was 64 (interquartile range [IQR] 59–70) years. A total of 180 patients underwent R0 resection, 4 patients underwent R2 resection, and 1 patient underwent R1 resection. All patients with R1/2 resection were included in the low-CONUT group. The clinicopathological characteristics of the two CONUT groups are shown in Table 2. The high-CONUT group was significantly associated with male sex (*p* = 0.046), lower BMI (*p* = 0.010), high-PLR (*p* < 0.001), high-NLR (*p* = 0.002), and higher GPS (*p* < 0.001).

**Table 2:** Relationships between clinical characteristics and the CONUT

Variables	Total (*n* = 185)		High-CONUT (*n* = 17)		Low-CONUT (*n* = 168)		*p* value
	n	%	n	%	n	%	
Age (years)							
< 65	95	51.4	9	52.9	86	51.2	
≥ 65	90	48.6	8	47.1	82	48.8	0.891
Sex							
Male	152	82.2	17	100	135	80.4	
Female	33	17.8	0	0	33	19.6	0.046*
BMI							
< 21.0	95	51.4	14	82.4	81	48.2	
≥ 21.0	90	48.6	3	17.6	87	51.8	0.010*
PS							
0	173	93.5	15	88.2	158	94.0	
1/2	12	6.5	2	11.8	10	6.0	0.304*
ASA score							
1	34	18.4	2	11.8	32	19.0	
2	140	75.7	14	82.4	126	75.0	
3	11	5.9	1	5.9	10	6.0	0.757
Location							
Upper	27	14.6	3	17.6	24	14.3	
Middle	106	57.3	8	47.1	98	58.3	
Lower	52	28.1	6	35.3	46	27.4	0.668
cT stage							
cT1	73	39.5	4	23.5	69	41.1	
cT2	44	23.8	5	29.4	39	23.2	
cT3	68	36.8	8	47.1	60	35.7	0.369
cN stage							
Negative	128	69.2	10	58.8	118	70.2	
Positive	57	30.8	7	41.2	50	29.8	0.331
Clinical TNM stage							
I	67	36.2	4	23.5	63	37.5	
II	78	42.2	7	41.2	71	42.3	
III/IV	40	21.6	6	35.3	34	20.2	0.293
SCCA (ng/ml)							
<2.0	148	80	12	70.6	136	81.0	
≥2.0	37	20	5	29.4	32	19.0	0.309
PLR							
High (>193)	32	17.3	10	58.8	22	13.1	
Low (≤193)	153	82.7	7	41.2	146	86.9	<0.001
NLR							
High (>3.612)	22	11.9	6	35.3	16	9.5	
Low (≤3.612)	163	88.1	11	64.7	152	90.5	0.002
GPS							
0	171	92.4	12	70.6	159	94.6	
1	13	7.0	4	23.5	9	5.4	
2	1	0.5	1	5.9	0	0	<0.001
Components of the CONUT score							
Albumin score							
0	183	98.9	15	88.2	168	100	
2/4	2	1.1	2	11.8	0	0	0.008*
TLC score							
0	125	67.6	0	0	125	74.4	
1	39	21.1	4	23.5	35	20.8	
2/3	21	11.4	13	76.5	8	4.8	<0.001
TC score							
0	113	61.1	2	11.8	111	66.1	
1	58	31.4	9	52.9	49	29.2	
2/3	14	7.6	6	35.3	8	4.8	<0.001

*CONUT* Controlling Nutritional Status, *BMI* body mass index, *PS* performance status

*ASA* American Society of Anesthesiology, *TNM* tumor-node-metastasis, *SCCA* squamous

cell carcinoma antigen, *PLR* platelet to lymphocyte ratio, *NLR* neutrophil to lymphocyte ratio

*GPS* Glasgow Prognostic Score, *TLC* total lymphocyte count, *TC* total cholesterol

*Fisher’s exact test

### Survival

The median follow-up period for survivors was 81.5 months (IQR 45.8–112.3 months). Three patients were lost to follow-up within 5 years, with the shortest follow-up period for survivors being 13 months. Recurrence was observed in 54 cases with a median duration to recurrence of 11 months (IQR 6.0–17.5 months). A total of 77 deaths were observed.

**Fig. 1:**
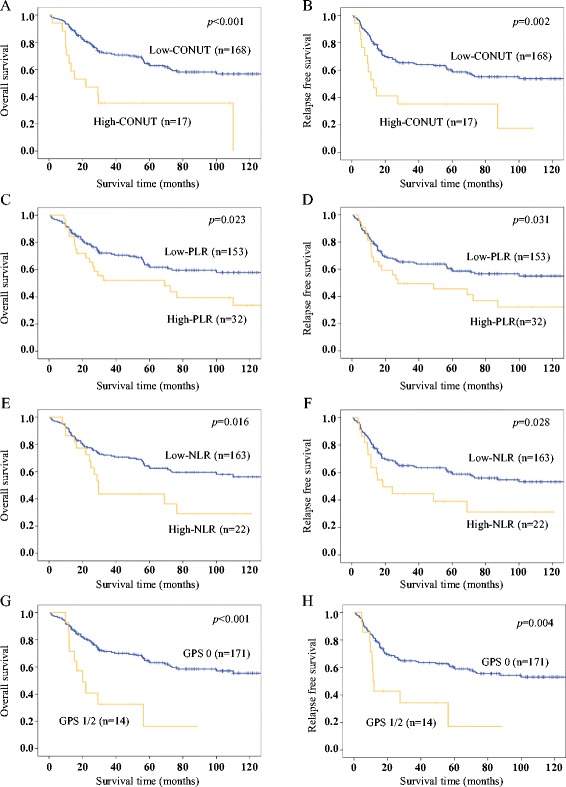
Kaplan-Meier survival curves of overall survival (OS) and relapse-free survival (RFS) in patients with resectable thoracic ESCC. **a** The 3- and 5-year OS rates are 72.0 % and 63.6 % in the low-CONUT group and 35.3 and 35.3 % in the high-CONUT group. **b** The 3- and 5-year RFS rates are 65.3 and 59.5 % in the low-CONUT group and 35.3 and 35.3 % in the high-CONUT group. **c** The 3- and 5-year OS rates are 72.1 and 62.6 % in the low-PLR group and 52.1 and 52.1 % in the high-PLR group. **d** The 3- and 5-year RFS rates are 65.4 and 59.5 % in the low-PLR group and 49.3 and 45.5 % in the high-PLR group. **e** The 3- and 5-year OS rates are 72.1 and 63.3 % in the low-NLR group and 43.5 and 43.5 % in the high-NLR group. **f** The 3- and 5-year RFS rates are 65.0 and 59.6 % in the low-NLR group and 44.6 and 39.0 % in the high-NLR group. **g** The 3- and 5-year OS rates are 71.4 and 63.9 % in the GPS 0 group and 32.7 and 16.3 % in the GPS 1/2 group. **h** The 3- and 5-year RFS rates are 64.8 and 59.8 % in the GPS 0 group and 34.3 and 17.1 % in the GPS 1/2 group

The 3- and 5-year OS and RFS rates for the entire study population were 68.5 and 60.7 %, and 62.6 and 57.1 %, respectively. The Kaplan-Meier survival curves comparing OS and RFS between two groups based on each nutritional and inflammation-based marker are shown in Fig. 1a-h. The OS and RFS rates were significantly lower in the high-CONUT (*p* < 0.001, *p* = 0.002), high-PLR (*p* = 0.023, *p* = 0.031), high-NLR (*p* = 0.016, *p* = 0.028), and GPS 1/2 (*p* < 0.001, *p* = 0.004) groups.

### Prognostic factors for OS and RFS

The results of univariate and multivariate analyses for OS and RFS are summarized in Tables 3 and 4. For the univariate analyses, the same factors, sex, PS, ASA, clinical TNM stage, CONUT score, PLR, NLR, and GPS, were significantly associated with OS and RFS. On multivariate analyses for OS and RFS using variables with *p* < 0.1 on univariate analyses and CONUT, PLR, NLR, GPS, or components of the CONUT score (serum albumin score, total lymphocyte count score, total cholesterol score), respectively, among the inflammation-based and nutritional markers, only the CONUT score was an independent predictive factor for OS (HR 2.303, 95 % CI 1.191–4.455; *p* = 0.013) and RFS (HR 2.163, 95 % CI 1.139–4.109; *p*–0.018).

**Table 3:** Univariate and multivariate analyses of prognostic factors for OS of patients with resectable thoracic ESCC

Variable	5-year OS (%)	Univariate	Multivariate	
		*p* value	HR (95 % CI)	*p* value
Total	60.7			
Age (years)		0.089	1.214 (0.753–1.956)	0.427
< 65	68.5			
≥ 65	52.1			
Sex		0.004	0.276 (0.107–0.711)	0.008
Male	56.0			
Female	83.3			
BMI		0.479		
<21.0	67.3			
≥21.0	54.2			
PS		<0.001	4.223 (2.155–8.274)	<0.001
0	64.6			
1/2	8.3			
ASA		<0.001		<0.001
1	81.2		1.000	
2	59.6		1.184 (0.554–2.528)	0.663
3	10.9		5.856 (2.102–16.310)	0.001
Location		0.941		
Upper	58.6			
Middle	60.7			
Lower	62.3			
cTNM stage		0.001		0.015
I	75.4		1.000	
II	57.5		1.515 (0.850–2.698)	0.159
III/IV	40.4		2.485 (1.338–4.615)	0.004
SCCA (ng/ml)		0.139		
<2.0	61.2			
≥2.0	59.6			
CONUT score		<0.001	2.303 (1.191–4.455)	0.013
High (≥3)	35.3			
Low (≤2)	63.6			
PLR		0.025	1.213 (0.696–2.115)	0.496
High (>193)	52.1			
Low (≤193)	62.6			
NLR		0.018	1.194 (0.627–2.273)	0.589
High (>3.612)	43.5			
Low (≤3.612)	63.3			
GPS		0.001	1.021 (0.465–2.245)	0.958
0	63.9			
1/2	16.3			
Components of the CONUT score				
Albumin score		0.012	1.096 (0.479–2.511)	0.828
0	61.4			
2/4	0			
TLC score		0.074	1.061 (0.518–2.171)	0.872
0/1	62.6			
2/3	47.6			
TC score		0.015	1.481 (0.688–3.190)	0.316
0/1	62.4			
2/3	40.8			

*BMI* body mass index, *PS* performance status, *ASA* American Society of Anesthesiology, *TNM* tumor-node-metastasis, *SCCA* squamous cell carcinoma antigen, *CONUT* Controlling Nutritional Status, *PLR* platelet to lymphocyte ratio, *NLR* neutrophil to lymphocyte ratio, *GPS* Glasgow Prognostic Score, *TLC* total lymphocyte count, *TC* total cholesterol

The results of multivariate analyses of age, sex, PS, ASA, cTNM stage in this table are the results of analyses with CONUT score

HRs and *p* values of PLR, NLR, GPS, Albumin score, TLC score, and TC score in this table are the results of respective multivariate analyses using variables with *p* < 0.1 on univariate analyses and each factor

**Table 4:** Univariate and multivariate analyses of prognostic factors for RFS of patients with resectable thoracic ESCC

Variable	5-year RFS (%)	Univariate	Multivariate	
		*p* value	HR (95 % CI)	*p* value
Total	57.1			
Age (years)		0.194		
< 65	63.2			
≥65	50.2			
Sex		0.014	0.458 (0.214–0.982)	0.045
Male	53.1			
Female	75.1			
BMI		0.394		
<21.0	62.6			
≥21.0	51.4			
PS		<0.001	4.029 (2.041–7.956)	<0.001
0	60.7			
1/2	8.3			
ASA		0.005		0.010
1	73.2		1.000	
2	56.2		1.117 (0.572–2.181)	0.746
3	10.9		3.600 (1.372–9.443)	0.009
Location		0.803		
Upper	57.5			
Middle	58.0			
Lower	55.0			
cTNM stage		<0.001		0.011
I	73.1		1.000	
II	52.5		1.731 (1.006–2.979)	0.048
III/IV	37.4		2.515 (1.361–4.646)	0.003
SCCA (ng/ml)		0.051	1.226 (0.717–2.097)	0.456
<2.0	59.0			
≥2.0	49.8			
CONUT score		0.003	2.163 (1.139–4.109)	0.018
High (≥3)	35.3			
Low (≤2)	59.5			
PLR		0.033	1.129 (0.656–1.943)	0.662
High (>193)	45.5			
Low (≤193)	59.5			
NLR		0.030	1.094 (0.569–2.102)	0.788
High (>3.612)	39.0			
Low (≤3.612)	59.6			
GPS		0.006	0.955 (0.439–2.078)	0.908
0	59.8			
1/2	17.1			
Components of the CONUT score				
Albumin score		0.012	1.334 (0.608–2.928)	0.472
0	57.7			
2/4	0			
TLC score		0.069	1.113 (0.551–2.250)	0.765
0/1	59.1			
2/3	42.9			
TC score		0.050	1.287 (0.604–2.743)	0.513
0/1	58.4			
2/3	41.7			

*BMI* body mass index, *PS* performance status, *ASA* American Society of Anesthesiology, *TNM* tumor-node-metastasis, *SCCA* squamous cell carcinoma antigen, *CONUT* Controlling Nutritional Status, *PLR* platelet to lymphocyte ratio, *NLR* neutrophil to lymphocyte ratio, *GPS* Glasgow Prognostic Score, *TLC* total lymphocyte count, *TC* total cholesterol

The results of multivariate analyses of sex, PS, ASA, cTNM stage, SCCA in this table are the results of analyses with CONUT score

HRs and *p* values of PLR, NLR, GPS, Albumin score, TLC score, and TC score in this table are the results of respective multivariate analyses using variables with *p* < 0.1 on univariate analyses and each factor

### Subgroup analysis

**Fig. 2:**
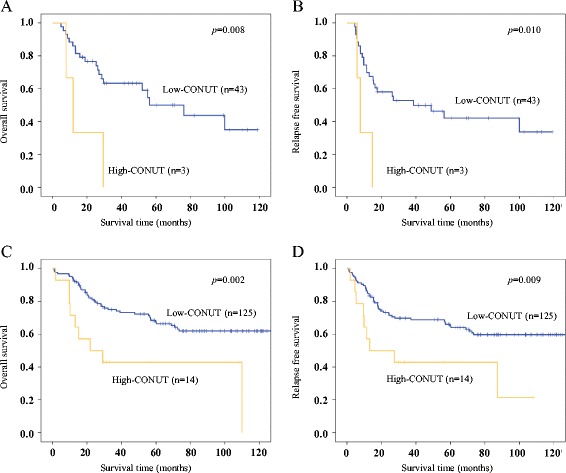
Kaplan-Meier survival curves of overall survival (OS) and relapse-free survival (RFS) according to the CONUT score in patients with preoperative treatment (**a** OS, *p* = 0.008; **b** RFS, *p* = 0.010) and in patients without preoperative treatment (**c** OS, *p* = 0.002; **d** RFS, *p* = 0.009)

A subgroup analysis according to the presence of preoperative treatment was conducted. In patients with or without preoperative treatment, the Kaplan-Meier survival curves comparing OS and RFS based on the CONUT score are shown in Fig. 2a-d. The OS and RFS rates were significantly lower in the high-CONUT group in patients with preoperative treatment (*p* = 0.008, *p* = 0.010) and in patients without preoperative treatment (*p* = 0.002, *p* = 0.009).

### Cause of death

Causes of death according to the CONUT score are shown in Table 5. The proportion of patients who died of primary disease was significantly higher in the high-CONUT group than in the low-CONUT group (47.1 % vs. 20.8 %, *p* = 0.015), whereas there were no significant differences in the rates of patients who died of other disease, other cancer, and postoperative complications.

**Table 5:** Cause of death

	Low-CONUT (*n* = 168)	High-CONUT (*n* = 17)	*p* value
Total	65 (38.7 %)	12 (70.6 %)	
Primary disease	35 (20.8 %)	8 (47.1 %)	0.015
Other disease	21 (12.5 %)	2 (11.8 %)	1.000*
Other cancer	7 (4.2 %)	1 (5.9 %)	0.545*
Postoperative complications	2 (1.2 %)	1 (5.9 %)	0.252*

*Fisher’s exact test

## Discussion

In the present study, the prognostic impacts of the CONUT score, PLR, NLR, and GPS were evaluated and compared in 185 patients with resectable thoracic ESCC. We found that only the CONUT score was an independent prognostic factor for OS and RFS, and it was superior to other inflammation-based markers in terms of predictive ability for prognosis before initial treatment.

The CONUT score was developed to assess nutritional status more easily and more objectively, having been validated in comparison with two other classical but slightly complicated assessment tools: the Subjective Global Assessment and the Full Nutritional Assessment [16]. The CONUT score is composed of the serum albumin concentration, total peripheral lymphocyte count, and total cholesterol concentration. The serum albumin concentration is known as a reliable indicator of nutritional status and systemic inflammation [20]. Total peripheral lymphocytes, which play an important role in the immune response to the tumor, are known to indicate the immunological and nutritional status. Total cholesterol concentration is known as an indicator of a patient’s caloric reserves [21]. Thus, a higher CONUT score could reflect not only malnutrition, but also systemic inflammation and an impaired immune response. Indeed, the high-CONUT group was significantly associated with lower BMI, high-PLR, high-NLR, and GPS1/2.

Recently, Hirahara et al. [22] first reported that the CONUT score was an independent predictor of cancer-specific survival in patients who underwent curative thoracoscopic esophagectomy for esophageal cancer. They also demonstrated that a high CONUT score was significantly associated with high NLR, and NLR was shown not to be a significant prognostic factor, consistent with the present results. The present study advances this previous study by determining the cut-off value using statistical methods, showing that the CONUT score is an independent prognostic factor for both OS and RFS and by comparing it with several inflammation-based markers.

The difference between the CONUT score and other inflammation-based markers is that the CONUT score includes the total cholesterol concentration in its calculation. Cholesterol is an essential component of the cell membrane that is involved in numerous biochemical pathways potentially correlated with cancer initiation and progression and the immune response. Several epidemiological studies and case control studies have demonstrated that a lower serum total cholesterol level was associated with increased mortality from several cancers [23–29]. Similarly, in the present study, a lower serum total cholesterol level was significantly associated with poorer survival. However, it remains uncertain whether hypocholesterolemia is a result of tumor progression or induces tumor progression. As a potential hypothesis to explain the relationship between hypocholesterolemia and cancer, a lowered serum cholesterol is attributed to the increased consumption of cholesterol needed for growth in cancer cells, which may indicate higher activity and malignancy of the tumor [25, 30]. This hypothesis could be supported by the fact that serum cholesterol levels increase after curative surgery and then decrease with cancer recurrence [31]. On the other hand, Calleros et al. [32] demonstrated that chronic cholesterol depletion induces NFkB activation, which could promote proliferation of malignant tumor cells. Muldoon et al. [33] reported that hypocholesterolemia was significantly more associated with fewer circulating lymphocytes, total T cells, and CD8+ cells than hypercholesterolemia. It was also reported that cholesterol increases the antigen-presenting function of monocytes [34]. Thus, a low serum total cholesterol level may contribute to a poorer prognosis by affecting intracellular signaling and impairing the immune system against tumor spread. Inclusion of the serum total cholesterol level in its calculation may be one of the reasons why the CONUT score is able to predict patients with a poorer prognosis more sensitively than other inflammation-based markers.

As in other malignancies, several studies have shown that PLR, NLR, and GPS were independent prognostic factors in esophageal cancer [11, 35]. In contrast, several studies did not show PLR and NLR to be independent prognostic factors [36–38]. In the present study, although PLR, NLR, and GPS were found to be predictive factors for OS and RFS on univariate analyses, multivariate analysis did not show these markers to be independent prognostic factors. These discrepancies may be due to the differences in sample size, follow-up periods, and cut-off values, which vary by method of determination and the population. Because the method to determine the optimal cut-off value has still not been fully established, various cut-off values have been used in previous studies [11, 14, 39]. In this study, the cut-off values to predict survival were determined from the maximal Youden indices based on the results of time-dependent ROC curve analyses [19, 40]. The present methodological approach seems to have advantages in terms of objectivity, but its validity has not been evaluated. Further studies are needed to establish the best method to determine cut-off values. The present study focused on resectable esophageal cancer patients who may have less systemic inflammation and better nutritional status than those with T4 and metastatic disease. Therefore, the proportions of high PLR and high NLR patients were lower than in previous reports [11, 41]. It might be important to identify an optimal cut-off value based on similar populations, such as tumor stage.

It is noteworthy that the CONUT score calculated before initial treatment was an independent prognostic factor, contributing to making an individual treatment strategy. Although the effects of perioperative nutritional intervention on the long-term outcome in patients with malnutrition due to malignant disease have not been confirmed, numerous studies have reported that perioperative nutritional intervention improved tolerance for anticancer treatment and reduced postoperative complications, which may contribute to improving the prognosis [42–44]. However, the definition and assessment of malnutrition in previous studies have not been unified. The CONUT score may be used as an index for selection of patients who need nutritional intervention and for evaluation of nutritional management in esophageal cancer treatment.

In the present study, the high-CONUT group was significantly associated with primary cancer death and poorer RFS. Similarly, Iseki et al. [17] and Hirahara et al. [22] reported that a higher CONUT score was an independent predictive factor for cancer-specific survival in colorectal cancer and esophageal cancer, respectively. These findings suggest that poorer nutritional status identified by the CONUT score may be involved in poorer tolerability for anticancer treatment and the growth of micrometastatic and residual cancer cells, which results in a worse prognosis. Patients with a high CONUT score may be candidates for not only nutritional intervention, but also more intensive multimodal treatment in resectable ESCC.

The present study has some limitations. First, this was a retrospective study conducted at a single institution, and the number of cases was limited. Second, potential factors that affect inflammation-based and nutritional markers, such as comorbidities and medications, could not be excluded. Third, there was heterogeneity in the neoadjuvant and adjuvant treatments in this study. However, in the subgroup analysis with or without neoadjuvant treatment, the high-CONUT group had significantly worse OS and RFS in both subgroups. Within these limitations, the present study demonstrated that the CONUT score is a promising prognostic factor with a better predictive value than PLR, NLR, and GPS in patients with resectable thoracic ESCC. Large-scale prospective validation studies are needed to confirm these findings.

## Conclusion

The CONUT score was found to be an independent predictor of OS and RFS before treatment, and it was superior to PLR, NLR, and GPS in terms of predicting prognosis in patients with resectable thoracic ESCC. The estimation of the CONUT score is inexpensive and easily available from laboratory data in daily clinical practice. We suggest that the CONUT score should be calculated routinely before initial treatment, and it could be a useful indicator for pretreatment nutritional management in esophageal cancer.

## Abbreviations

ASA: American Society of Anesthesiology score
AUC: Areas under the curve
BMI: Body mass index
CI: Confidence interval
CONUT: Controlling nutritional status
ESCC: Esophageal squamous cell carcinoma
GPS: Glasgow prognostic score
HR: Hazard ratio
IQR: Interquartile range
NLR: Neutrophil to lymphocyte ratio
OS: Overall survival
PLR: Platelet to lymphocyte ratio
PS: Eastern cooperative oncology group performance status
RFS: Relapse-free survival
ROC: Receiver operating characteristic
SCCA: Serum squamous cell carcinoma antigen
